# Inhibition of Vascular Smooth Muscle Cell Proliferation by ENPP1: The Role of CD73 and the Adenosine Signaling Axis

**DOI:** 10.3390/cells13131128

**Published:** 2024-06-29

**Authors:** Boris Tchernychev, Yvonne Nitschke, Di Chu, Caitlin Sullivan, Lisa Flaman, Kevin O’Brien, Jennifer Howe, Zhiliang Cheng, David Thompson, Daniel Ortiz, Frank Rutsch, Yves Sabbagh

**Affiliations:** 1 Research and Development, Inozyme Pharma, 321 Summer St, Suite 400, Boston, MA 02201, USA; boris.tchernychev@inozyme.com (B.T.); di.chu@inozyme.com (D.C.); caitlin.sullivan@inozyme.com (C.S.); lisa.flaman@inozyme.com (L.F.); kevin.obrien@inozyme.com (K.O.); jennifer.howe@inozyme.com (J.H.); zlcheng2012@yahoo.com (Z.C.); david.thompson@inozyme.com (D.T.); daniel.ortiz@inozyme.com (D.O.); yves.sabbagh@inozyme.com (Y.S.); 2 Department of General Pediatrics, Münster University Children’s Hospital, 48149 Münster, Germany; yvonne.nitschke@ukmuenster.de; 3 INTEC Network of Ectopic Calcification, Center for Medical Genetics Ghent, Corneel Heymanslaan 10, 9000 Ghent, Belgium

**Keywords:** neointima, vascular smooth muscle cells, purinergic signaling

## Abstract

The Ectonucleotide Pyrophosphatase/Phosphodiesterase 1 (ENPP1) ectoenzyme regulates vascular intimal proliferation and mineralization of bone and soft tissues. *ENPP1* variants cause Generalized Arterial Calcification of Infancy (GACI), a rare genetic disorder characterized by ectopic calcification, intimal proliferation, and stenosis of large- and medium-sized arteries. ENPP1 hydrolyzes extracellular ATP to pyrophosphate (PP_i_) and AMP. AMP is the precursor of adenosine, which has been implicated in the control of neointimal formation. Herein, we demonstrate that an ENPP1-Fc recombinant therapeutic inhibits proliferation of vascular smooth muscle cells (VSMCs) in vitro and in vivo. Addition of ENPP1 and ATP to cultured VSMCs generated AMP, which was metabolized to adenosine. It also significantly decreased cell proliferation. AMP or adenosine alone inhibited VSMC growth. Inhibition of ecto-5′-nucleotidase CD73 decreased adenosine accumulation and suppressed the anti-proliferative effects of ENPP1/ATP. Addition of AMP increased cAMP synthesis and phosphorylation of VASP at Ser157. This AMP-mediated cAMP increase was abrogated by CD73 inhibitors or by A_2a_R and A_2b_R antagonists. Ligation of the carotid artery promoted neointimal hyperplasia in wild-type mice, which was exacerbated in ENPP1-deficient *ttw/ttw* mice. Prophylactic or therapeutic treatments with ENPP1 significantly reduced intimal hyperplasia not only in *ttw/ttw* but also in wild-type mice. These findings provide the first insight into the mechanism of the anti-proliferative effect of ENPP1 and broaden its potential therapeutic applications beyond enzyme replacement therapy.

## 1. Introduction

VSMCs are major constituents of the vascular wall and are critical for its integrity and in regulation of blood flow. In a healthy vessel, VSMCs are quiescent, and their growth is tightly controlled. Under various pathological conditions, quiescent VSMCs alter their phenotype and become highly proliferative. Dysregulated proliferation of smooth muscle cells leads to the progressive narrowing of the vessel lumen and drives the pathogenesis of many vaso-occlusive diseases and conditions. Hyperproliferation of pulmonary artery VSMCs promotes vascular remodeling and muscularization of small arteries in patients with pulmonary artery hypertension [1] and COPD [2]. Vascular smooth muscle cell hyperplasia contributes to the growth of the occlusive lesions seen in patients with Moyamoya disease [3]. Renal failure is a recognized risk factor for pathological vascular remodeling and venous intimal hyperplasia is a major cause of vascular access failure in patients with advanced chronic kidney disease and end-stage renal disease (ESRD) who require hemodialysis [4]. Moreover, in ESRD, advanced preexisting venous neointima was also observed prior to dialysis access procedures [5,6]. Calcific uremic arteriolopathy (calciphylaxis) is a rare complication of ESRD which affects small arteries. It is characterized by medial calcification, complete obstruction of subcutaneous arterioles by thrombi and by proliferating arterial smooth muscle cells that cause necrosis of dermal tissues [7]. Intimal hyperplasia is also a known complication of vascular and surgical procedures that include arterial bypass surgery, angioplasty, stenting, and transplantation. Excessive proliferation of smooth muscle cells induced by surgery or vascular intervention facilitates vein graft failure [8], in-stent restenosis [9], and cardiac allograft vasculopathy [10].

Generalized Arterial Calcification of Infancy (GACI) is a rare genetic disorder characterized by calcification and narrowing of medium-sized and large arteries [11]. GACI patients develop serious cardiovascular morbidities as a result of vascular calcification and arterial stenosis. These include hypertension and myocardial ischemia, which can lead to congestive heart failure and stroke [12]. More than 70% of patients affected by GACI carry pathogenic variants in the *ENPP1* gene [12]. ENPP1 is a type II transmembrane glycoprotein expressed in many tissues [13]. It belongs to the Ectonucleotide Pyrophosphatase/Phosphodiesterase (ENPP) family of proteins [13]. ENPP1 catalyzes hydrolysis of extracellular ATP to produce AMP and inorganic pyrophosphate (PP_i_). Pyrophosphate inhibits formation of calcium hydroxyapatite crystals and functions as an endogenous inhibitor of mineralization [14]. Variants in the *ENPP1* gene have been linked to abnormal calcification of soft and skeletal tissues in ectopic mineralization disorders, which include GACI, Cole disease, autosomal recessive hypophosphatemic rickets type II (ARHR2), and ossification of the posterior longitudinal filament of the spine (OPLL) [15,16]. In addition to its role in control of mineralization, ENPP1 has also been implicated in the regulation of VSMC proliferation. In fact, arterial stenosis associated with GACI is caused by proliferation of intimal smooth muscle cells [17]. Clinical observations in patients have been replicated in recent preclinical studies. Animal models of vascular injury and cell-based assays have shown that ENPP1 deficiency is associated with increased VSMC proliferation [18,19]. This accelerated VSMC growth was abolished by treatment with recombinant ENPP1-Fc fusion protein, AMP, and adenosine [18]. It has been proposed that the AMP that is generated by ENPP1 hydrolysis of ATP inhibits pathological growth of VSMCs [18]. In the current study, the mechanisms underlying the anti-proliferative effect of recombinant ENPP1 and AMP were investigated. In vitro assays with primary VSMCs treated with ENPP1 indicated that extracellular AMP plays an important role in the control of VSMC proliferation. Moreover, it was found that the adenosine produced by AMP hydrolysis, which is mediated by the endogenous 5′-nucleotidase CD73, was necessary for this effect. Adenosine participated in activation of signaling pathways implicated in vaso-protection. Extension of these studies revealed that prophylactic and therapeutic treatments of wild-type and ENPP1-deficient mice with recombinant ENPP1 significantly attenuated carotid artery ligation induced intimal hyperplasia.

## 2. Materials and Methods

### 2.1. Animals, Cells and Reagents

ENPP1-deficient *ttw/ttw mice* and wild-type (WT) littermate controls in a C57BL/6 genetic background were generated by heterozygous mating. The pups were weaned at 3–4 weeks of age and then maintained on a normal chow diet. *Enpp1* genotyping was performed using polymerase chain reaction analysis of tail DNA as described previously [20]. Primary VSMCs isolated from human aorta were purchased from Cell Applications Inc. (San Diego, CA, USA). Complete smooth muscle cell growth medium (SmGM-2) BulletKit and smooth muscle cell basal medium (SmBM) were from Lonza (Bend, OR, USA). Smooth muscle cell differentiation media were obtained from Cell Applications Inc. (San Diego, CA, USA), CisBio cAMP Gs dynamic kit and Phospho-VASP (Ser157) cellular kit were from Perkin Elmer Inc. (Waltham, MA, USA). Adenosine, inosine, AMP, and a Roche Chemiluminescent BrdU ELISA kit were from Sigma-Aldrich (St. Louis, MO, USA). ATP, PKA inhibitor KT5720, A_2a_R antagonist SCH-58261, and A_2b_R antagonist PBS-1115 were from Tocris Biosciences (Minneapolis, MN, USA). CD73 inhibitor AB680 and dual A_2a_R/A_2b_R antagonist AB928 were obtained from MedChemExpress (Monmouth Junction, NJ, USA). Antibodies to SM-MHC, SM-Calponin, CD39, and house-keeping proteins including GAPDH and α tubulin were purchased from Thermo Fisher Scientific (Waltham, MA, USA). Two forms of recombinant ENPP1 protein were used: INZ-701 [21] and IMA2a [22]. Both forms have identical enzymatic activity and are comprised of the extracellular portion of human ENPP1 that is linked to the *N*-terminus of a human IgG1 Fc domain. In comparison to INZ-701, IMA2a has an introduced FcRn binding site in the Fc domain and an additional N-linked glycosylation site in the catalytic domain of ENPP1. INZ-701 and IMA2a were produced in CHO cells and were purified using protein A affinity chromatography.

### 2.2. mRNA and Western Blot Analysis in Contractile and Synthetic Smooth Muscle Cells

Synthetic VSMCs were cultured in complete growth medium. To differentiate cells to contractile phenotype VSMCs were cultured in differentiation medium for 10 days. Medium was changed every two to three days. Total RNA was extracted from synthetic and contractile human aortic smooth muscle cells using PureLink^TM^ RNA mini kit (Thermo Fisher Scientific, Waltham, MA, USA) and Homogenizer (Thermo Fisher Scientific, Waltham, MA, USA). The isolated RNA was quantified using Agilent 2100 Bioanalyzer and reverse transcribed into cDNA using SuperScript^TM^ IV VILO^TM^ Master Mix with ezDNase^TM^ Enzyme (Thermo Fisher Scientific, Waltham, MA, USA). The resulting cDNA was amplified using the TaqMan^TM^ Fast Advanced Master Mix (Thermo Fisher Scientific, Waltham, MA, USA) and detected by real-time PCR using a QuantStudio^TM^ 5 Real-Time PCR System. TaqMan probes for human SM-MHC (MYH10, Hs00992055_m1), SM-Calponin (CNN1, Hs00154543_m1), ENPP1 (Hs01054038_m1), CD39 (ENTPD1, Hs00969559_m1), CD73 (NT5E, Hs01573922_m1), adenosine A_1_ receptor (ADORA1, Hs00181231_m1), adenosine A_2a_ receptor (ADORA2A, Hs00169123_m1), adenosine A_2b_ receptor (ADORA2B, Hs00386497_m1), and a housekeeping gene, GAPDH (Hs99999905_m1) were obtained from Thermo Fisher Scientific (Waltham, MA, USA). The target gene expression level was normalized by the GAPDH level in each sample. For Western blot analysis, whole cell lysates were prepared from synthetic and contractile HAOSMCs using RIPA Lysis and Extraction Buffer (Thermo Fisher Scientific, Waltham, MA, USA) with Halt Protease and Phosphate Inhibitor Cocktail (Thermo Fisher Scientific, Waltham, MA, USA). The protein concentration was measured using the Pierce BCA Protein Assay kit (Thermo Fisher Scientific, Waltham, MA, USA), and 20 µg of total protein was subjected to Western blotting. The protein was separated on 4–15% Mini-PROTEAN TGX Stain-Free Protein Gels (Bio-Rad, Hercules, CA, USA) and subsequently transferred onto PVDF membranes. Membranes were blocked with Intercept (TBS) Blocking Buffer (LI-COR, Lincoln, NE, USA) at room temperature for one hour and then incubated with primary antibodies followed by the incubation with the species-specific secondary antibodies (LI-COR, Lincoln, NE, USA). Bands were visualized using the LI-COR Odessey CLx system (LI-COR, Lincoln, NE, USA).

### 2.3. Cell-Based Assays

*Proliferation assay:* Cell proliferation was quantified by BrdU incorporation. Briefly, VSMCs (passage 4–5) were seeded at 2500 cell/well in white 96-well plateDelta surface, Nunc (Thermo Fisher Scientific, Waltham, USA) and cultured for 48 h in complete growth medium. Cells were starved for 24 h in basal medium. The next day, cells were stimulated with basal medium supplemented with heat-inactivated FBS (5%). Adenosine, inosine, ATP, AMP, ENPP1, and CD73 inhibitor AB680 were added to medium at indicated concentrations. Cells were grown for 72 h. Cultured medium with reagents was replaced daily. BrdU was added during the last 18 h of culture.

*cAMP assay:* VSMCs were plated on collagen I-coated 48-well plate (STEMCELL Technologies Inc., Vancouver, BC, Canada) at 100,000 cells/well and grown for 48 h in complete growth medium. Then cells were pretreated for 18 h with the inhibitor of CD73 AB680 (1 µM) or inhibitors of adenosine receptors including SCH-58261 (1 µM), PSB-1115 (1 µM), and AB928 (0.3 µM). Cells were washed once with basal medium and incubated for 4 h in basal medium containing AMP (30, 100 µM) in the absence or presence of inhibitors of adenosine receptors or CD73. Total cAMP levels were measured using the HTRF cAMP Gs dynamic kit (PerkinElmer, Waltham, MA, USA).

*p-VASP^Ser157^ assay:* VSMCs were plated on collagen I-coated 96-well plate at 50,000 cells/well in complete growth medium and cultured for 48 h. Cells were washed once with basal medium and incubated for 30 min in basal medium supplemented with FBS (0.25%) in the presence or absence of PKA inhibitor KT5720 (5 µM). Next, cells were stimulated for 30 min with basal medium containing AMP (30 or 100 µM) in the absence or presence of KT5720. The phosphorylation of VASP at Ser 157 was determined using the HTRF Phospho-VASP (Ser157) cellular kit (PerkinElmer, Waltham, MA, USA).

### 2.4. Analysis of Adenosine Metabolites

VSMCs were cultured in complete growth medium. After reaching 80% confluency, cells were plated on a collagen I-coated 96-well plate at 2500 cells/well in complete growth medium and cultured for 48 h, followed by 24 h of serum starvation. Cells were washed and incubated in basal medium containing ATP (0.3 mM) in the absence or presence of recombinant ENPP1 (0.2 µg/mL). At the indicated time points culture medium was collected, and 3M chilled formic acid was added to medium samples at a 1:1 ratio. The amounts of AMP, adenosine, and inosine in the culture medium were measured using HPLC.

### 2.5. Carotid Artery Ligation Model

All animal studies were approved by the local committee for animal studies and were performed according to the guidelines from Directive 2010/63/EU of the European Parliament on the protection of animals used for scientific purposes. Briefly, left carotid artery ligation surgery was performed in 7–8-week-old WT and *ttw/ttw* mice of both sexes. Mice were anesthetized by isoflurane inhalation and left carotid arteries were exposed through a small midline incision in the neck and ligated by a 5–0 silk suture about 2 mm proximal from the carotid bifurcation. Wounds were closed with resorbable 5–0 Vicryl suture and disinfected using Polyvidone-iodine solution. After surgery, mice received injections of Carprofene for 3 days as analgesic (0.5 mg/kg, s.c.). The ENPP1-Fc fusion protein, INZ-701, was used to evaluate the effect of treatment on intimal hyperplasia. INZ-701 (10 mg/kg) or vehicle (Tris buffered saline, pH 7.4) were administered subcutaneously every other day. In the prophylactic study, administration started 7 days prior to carotid artery ligation and treatment continued for 14 days post surgery. In the therapeutic study, treatment with INZ-701 started on day 7 after ligation and continued for 7 days. On day 14, after surgery mice were euthanized and perfused with 4% PFA in PBS, perfusion-fixed carotid arteries were dissected and embedded in paraffin blocks for further analysis.

### 2.6. Histomorphometry and Immunohistochemistry

Serial transverse 5 μm sections of the left carotid artery were prepared starting at the ligation site and ending at not more than 1 mm caudally from the ligation site. For morphometric analysis, sections were stained for elastic fibers using Elastica von Gieson staining (Roth, Karlsruhe, Germany). Morphometric analysis was performed in a blinded fashion on every fifth section (every 25 μm) located caudally from the ligation site, for a total of 12 sections per animal, spanning a distance of approximately 300 μm. The intimal and medial areas were measured using NIS Elements Imaging Software Version 5.11.01 (Nikon Instruments Europe, Amsterdam, The Netherlands). The areas within the circumferences of the external elastic lamina, the internal elastic lamina, and the luminal border were measured. The medial area, the intimal area, and the intima/media ratio (I/M ratio) were then calculated, whereas the area between external elastic lamina and internal elastic lamina displays the medial area, and the area between internal elastic lamina and luminal boarder displays the intimal area. The intimal and medial area values represent means for 12 sections for each mouse. For VSMCs proliferation analysis, PFA-fixed sections were stained with Ki-67 (Novus Biologicals LLC., Centennial, CO, USA) and αSMA specific antibodies (Cell Signaling Technology Inc., Danvers, MA, USA).

### 2.7. Statistical Analysis

Statistical analysis was performed using *t*-test. Comparisons of multiple groups used one-way ANOVA, followed by the Bonferroni’s post hoc test, performed with GraphPad Prism software version 8. *p* values of *p* < 0.05 were considered significant.

## 3. Results

### 3.1. Expression of Ectonucleotidases and Adenosine Receptors in Contractile and Synthetic VSMCs

**Figure 1:**
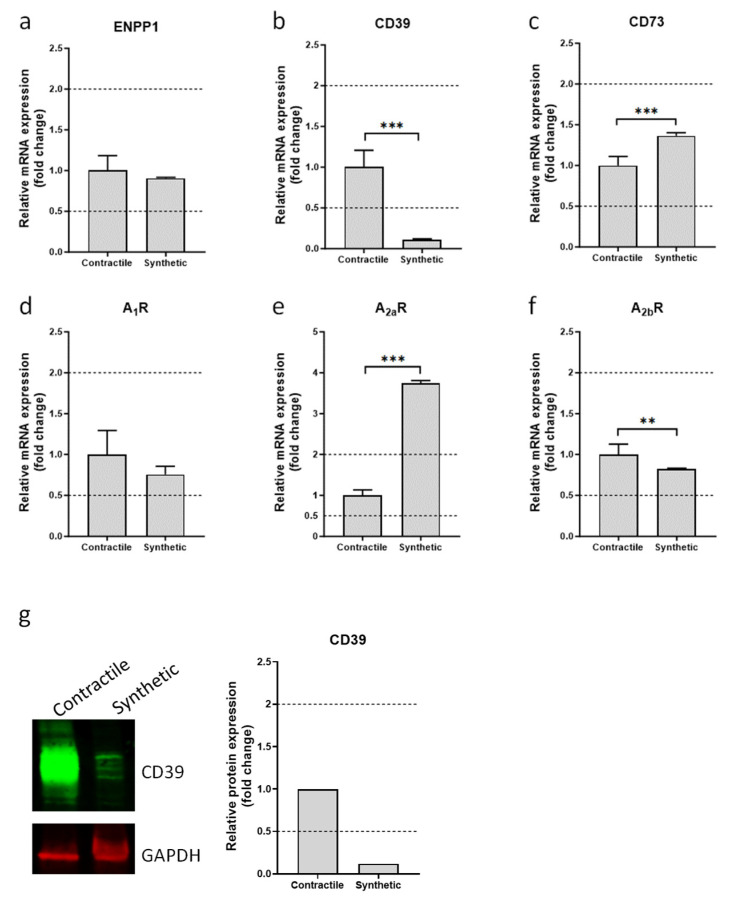
Analysis of ectonucleotidases and adenosine receptors mRNA in contractile and synthetic VSMCs. VSMCs were differentiated towards synthetic or contractile phenotypes. Expression of mRNA encoding ENPP1 (**a**), ATP/ADP-specific ectonucleotidase CD39 (**b**), AMP-specific ectonucleotidase CD73 (**c**), and A_1_, A_2a_, A_2b_ adenosine receptors (**d**–**f**) was analyzed using qPCR. Expression of CD39 protein in contractile and synthetic VSMCs (**g**). Values are presented as the mean ± SD, *n* = 6, ** *p* < 0.01, *** *p* < 0.001 (*t*-test).

Neointima formation is characterized by a profound change in the VSMC phenotype from a healthy, contractile, quiescent state to a pathologic, synthetic, proliferative state. To study the effect of phenotypic switching on the expression of ectonucleotidases and adenosine receptors, primary VSMCs were differentiated towards synthetic and contractile phenotypes. Compared to the cells differentiated towards a synthetic phenotype, VSMCs differentiated towards a contractile phenotype exhibited an increased expression of contractile markers that included smooth muscle myosin heavy chain and smooth muscle calponin (Supplementary Figure S1a). Increased proliferation is one of the hallmarks of synthetic VSMCs [23]. Therefore, cell growth was measured to confirm the phenotypic switch in a functional assay. In line with the gene expression analysis, VSMCs that differentiated towards the synthetic phenotype demonstrated a marked increase in proliferation when compared to contractile VSMCs (Supplementary Figure S1b). Gene expression analysis of ectonucleotidases revealed that the levels of ENPP1 and CD73 mRNAs were similar in synthetic and contractile VSMCs. However, expression of CD39 mRNA was significantly lower in synthetic VSMCs (Figure 1). Since CD39 is a major ectonucleotidase expressed in the vascular wall, the change in its expression was further confirmed by Western blot analysis (Figure 1). Analysis of mRNAs encoding adenosine receptors demonstrated that expression of the A_2a_ adenosine receptor was significantly higher in synthetic VSMCs (Figure 1), while levels of A_1_ and A_2b_ receptors mRNAs were similar in contractile and synthetic VSMCs (Figure 1). No A_3_ receptor mRNA was detected in VSMCs of either phenotype.

### 3.2. Effect of ENPP1/ATP, AMP, and Adenosine on Proliferation of VSMCs

**Figure 2:**
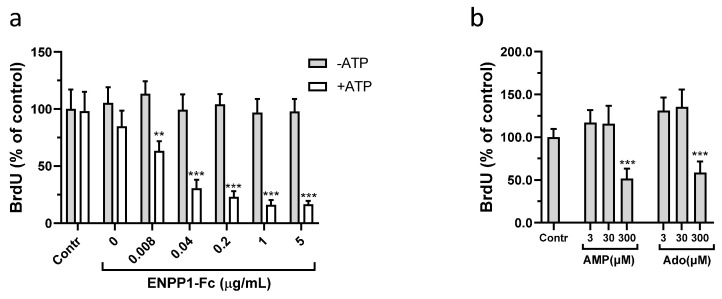
Effect of ENPP1—Fc, AMP, and adenosine (Ado) on VSMCs proliferation. Synthetic VSMCs were starved for 24 h in basal media. Cells were then cultured for 72 h in FBS (5%) containing media in the presence or absence of ENPP1—Fc/ATP (**a**), 300 μM ATP alone (**a**), AMP or adenosine (**b**). Cell proliferation was evaluated by BrdU incorporation. Values are presented as the mean ± SD, *n* = 8. ** *p* < 0.01, *** *p* < 0.001 (one-way ANOVA).

Previously, it was shown that iPSC-derived human VSMC proliferation increased following silencing of ENPP1 expression. Conversely, treatment with ENPP1-Fc and ATP together or AMP or adenosine alone inhibited proliferation of these cells [18]. In the present study, treatment of synthetic VSMCs with ATP combined with increasing concentrations of exogenous ENPP1 significantly inhibited cell proliferation (Figure 2a). Addition of ATP or of ENPP1 alone had no effect. These results suggest that the enzymatic hydrolysis of ATP by ENPP1 to form AMP is required for the anti-proliferative effect of the ENPP1 and ATP treatment. Support of this hypothesis comes from the finding that VSMC proliferation was reduced by treatment of cells with AMP alone, or by treatment with adenosine, which is a breakdown product of AMP (Figure 2b).

### 3.3. Role of CD73 in Antiproliferative Effect of ENPP1/ATP

**Figure 3:**
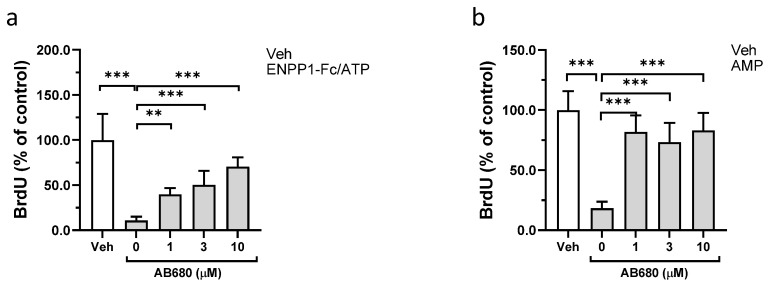
Role of CD73 in antiproliferative effect of ENPP1-Fc/ATP and AMP. Synthetic VSMCs were starved for 24 h in serum-free media. Then cells were cultured in a media supplemented with heat-inactivated FBS (5%) in the presence of AB680 and, either (**a**) ENPP1-Fc (0.2 μg/mL) and ATP (300 μM) or (**b**) AMP (300 μM). After 3 days, cell proliferation was evaluated by BrdU incorporation. Values are presented as the mean ± SD, *n* = 8, ** *p* < 0.01, *** *p* < 0.001, (one-way ANOVA).

Extracellular AMP is hydrolyzed to adenosine by the membrane-bound ectonucleotidase CD73. To investigate the role that CD73 might play in the anti-proliferative effect of ENPP1/ATP and of AMP, cells were treated with the potent and selective CD73 inhibitor AB680. In a control experiment, AB680 has no effect on the enzymatic activity of recombinant ENPP1 (Supplementary Figure S2). Pharmacological inhibition of CD73 with AB680 significantly attenuated the antiproliferative effect of the ENPP1/ATP treatment (Figure 3a). Likewise, treatment with AB680 also reversed the effect of AMP on VSMCs proliferation (Figure 3b).

**Figure 4:**
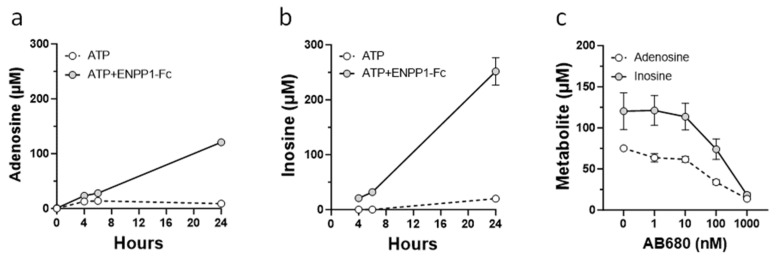
Metabolism of extracellular AMP by VSMCs. Synthetic VSMCs were incubated in basal media supplemented with 300 μM ATP (**a**,**b**) or with ENPP1-Fc (0.2 μg /mL) and ATP (**a**–**c**) in the presence or absence of CD73 inhibitor AB680 (**c**). Concentrations of adenosine and inosine in culture media were determined by HPLC. Values are presented as the mean ± SD, *n* = 6–8.

To confirm that synthetic VSMCs can metabolize extracellular AMP to adenosine, culture medium collected from cells treated with ENPP1 and ATP or with ATP alone was analyzed. Since extracellular adenosine is unstable and can be further degraded by adenosine deaminase to the more stable metabolite inosine, analysis of inosine was also included. Treatment of synthetic VSMCs with ENPP1 and ATP resulted in accumulation of adenosine and inosine in culture medium (Figure 4a,b). In contrast, cells treated with ATP alone showed only a limited increase in adenosine and inosine in the medium (Figure 4a,b) and inhibition of CD73 with AB680 completely abolished accumulation of adenosine and inosine in VSMC medium (Figure 4c).

Synthetic VSMCs treated with adenosine alone exhibited a marked decrease in cell growth (Figure 2b). Because a significant accumulation of inosine was observed in the medium from ENPP1/ATP-treated cells, the effect of inosine on cell growth was examined. Inosine had no effect on VSMCs proliferation (Supplementary Figure S3), suggesting that the anti-proliferative effects observed with either ENPP1/ATP, or AMP, treatments were mediated by generation of adenosine alone.

### 3.4. Activation of cAMP-PKA Signaling Pathway by Extracellular AMP

**Figure 5:**
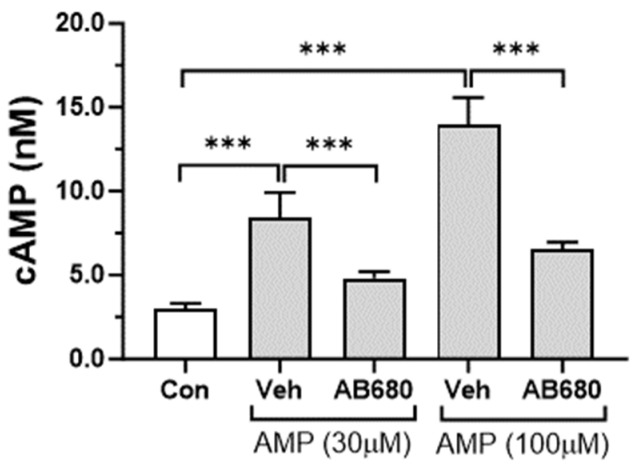
Effect of extracellular AMP and CD73 inhibition on cAMP levels in VSMCs. Synthetic VSMCs were pretreated for 18 h with vehicle (Veh) or CD73 inhibitor AB680 (1 μM). Cells were washed and stimulated for 4 h with AMP in the presence of vehicle or AB680. Total cAMP was measured using HTRF assay. Values are presented as the mean ± SD, *n* = 6, *** *p* < 0.001 (*t*-test).

The two adenosine receptors that were shown to be expressed in VSMCs were Gs-coupled GPCRs. Downstream signaling for these receptors is mediated by the cAMP second messenger. Therefore, cAMP synthesis was examined in cells stimulated with AMP. Treatment with AMP increased cAMP levels in synthetic VSMCs (Figure 5). This effect was completely abolished by the CD73 inhibitor AB680, suggesting that conversion of extracellular AMP to adenosine is required for the AMP-stimulated cAMP increase (Figure 5).

**Figure 6:**
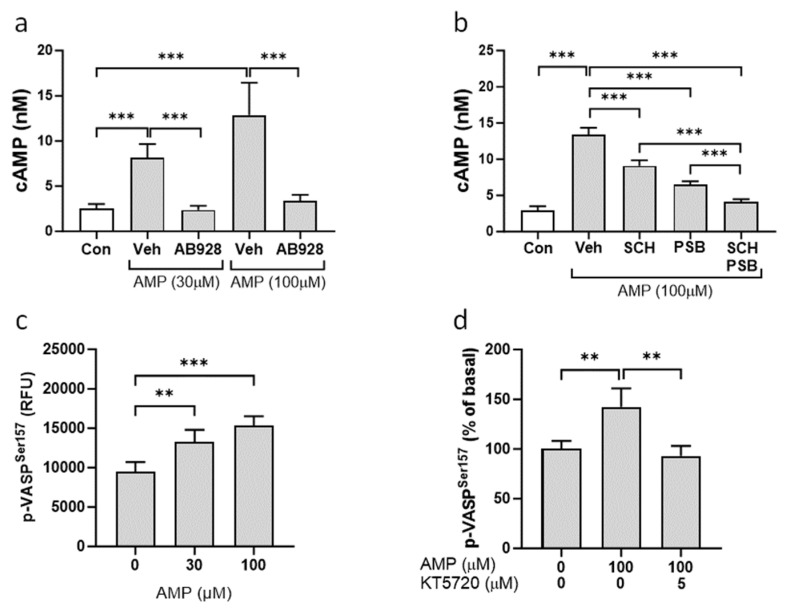
Effect of AMP on activation of Gs-coupled adenosine receptors and PKA. Synthetic VSMCs were pretreated for 18 h with either (**a**) dual A_2a_R/A_2b_R antagonist AB928 (0.3 μM) or (**b**) selective A_2a_R antagonist SCH-58261 (1 μM) and selective A_2b_R antagonist PSB-1115 (1 μM). Cells were washed and stimulated for 4 h with AMP in the presence or absence of inhibitors. Total cAMP was measured using HTRF assay. VSMCs were preincubated for 30 min in the basal media supplemented with 0.25% FBS and stimulated for 30 min with AMP in the absence (**c**) or presence (**d**) of PKA inhibitor KT5720. Phosphorylation of VASP at Ser157 was analyzed using HTRF assay. Values are presented as the mean ± SD, *n* = 4–6, ** *p* < 0.01, *** *p* < 0.001 (*t*-test).

To examine whether the extracellular adenosine that is generated from AMP exerts its effect on cAMP synthesis through activation of Gs-coupled adenosine receptors, cells were treated with the dual A_2a_R/A_2b_R antagonist AB928. Concurrent inhibition of A_2a_R and A_2b_R by AB928 abrogated the cAMP increase produced by AMP treatment (Figure 6a). Specific inhibition of the A_2a_ and A_2b_ receptors using the SCH-58261 and PSB-1115 antagonists, respectively, also reduced cAMP levels in VSMCs (Figure 6b). Moreover, the SCH-58261 and PSB-1115 inhibitors had an additive effect on cAMP synthesis, which was similar to the effect of the dual A_2a_R/A_2b_R antagonist AB928 (Figure 6b). Protein kinase A (PKA) is one of the main targets for intracellular cAMP, which acts as a co-factor that triggers the kinase activity of PKA. The activation of PKA was assessed by examining phosphorylation of its substrate, vasodilator-stimulated phosphoprotein (VASP), at serine 157. Treatment of VSMCs with AMP increased phosphorylation of VASP at serine 157 (Figure 6c). Conversely, incubation of cells with the PKA inhibitor KT5720 significantly reduced AMP-mediated VASP phosphorylation (Figure 6d).

### 3.5. In Vivo Effect of ENPP-Fc Fusion Protein INZ-701 on Neointima Formation

**Figure 7:**
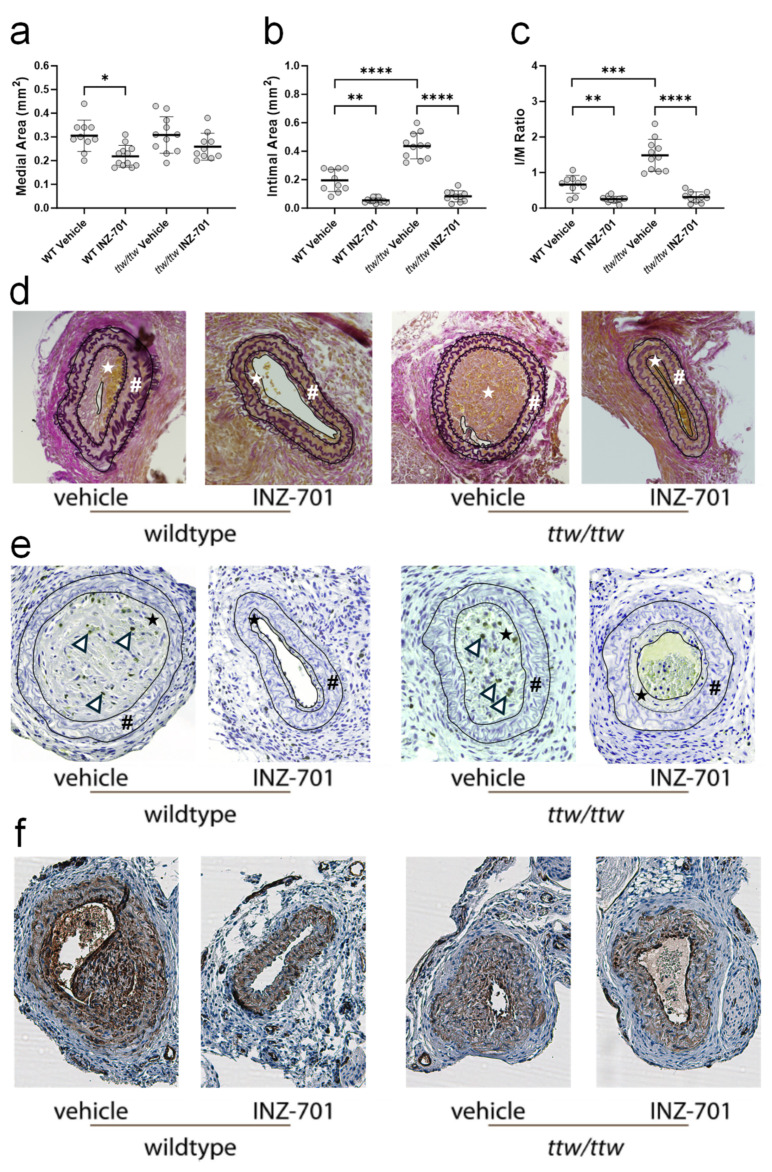
Effect of prophylactic treatment with INZ-701 on neointima formation in WT and *ttw/ttw* mice. Treatment of mice with either vehicle or INZ-701 (10 mg/kg) started 7 days before ligation of left carotid artery. Carotid arteries were collected on day 14th after ligation and fixed on formalin. Morphometric analysis of elastin-stained sections was performed to determine medial (**a**) and intimal (**b**) areas, and the intima/media ratio (**c**). Elastica von Gieson’s staining of carotid arteries cross sections from vehicle or INZ-701-treated WT and *ttw/ttw* mice (**d**). Smooth muscle cell proliferation was assessed by immunohistochemical staining of Ki-67 (arrowhead) (**e**). From outside to inside, external elastic lamina, internal elastic lamina, and luminal boarder (if existing) are circled for better visualization in (**d**,**e**). The area between external elastic lamina and internal elastic lamina displays the medial area (#), the area between internal elastic lamina and luminal boarder displays the intimal area (star). Staining of alpha smooth muscle actin (**f**) Values are presented as the mean ± SD, *n* = 10–12, * *p* < 0.05, ** *p* < 0.01, *** *p* < 0.001, **** *p* < 0.0001 (one-way ANOVA).

A carotid ligation model was chosen to study the impact of ENPP1-Fc treatment on VSMC proliferation in wild-type (WT) and ENPP1-deficient tiptoe walking (*ttw/ttw*) mice. Treatment of wild-type and *ttw/ttw* mice with either vehicle or INZ-701 was initiated 7 days prior to carotid ligation. Treatment continued for 14 days post ligation. Two weeks after surgery, WT and ttw/ttw mice treated with ENPP1 showed greatly reduced medial area (Figure 7a), intimal area (Figure 7b), and I/M ratio (Figure 7c) compared to animals treated with vehicle. Intimal and medial areas, as well as the I/M ratio, of ENPP1 treated *ttw/ttw* mice approached the level observed in INZ-701-treated WT mice. Conversely, vehicle-treated *ttw/ttw* mice developed a significantly increased intimal area and I/M ratio compared to vehicle-treated WT mice (Figure 7b,c). Histological Elastica van Gieson staining of carotid sections from 14 days ligated mice revealed much reduced intimal hyperplasia in INZ-701-treated WT and *ttw/ttw* mice when compared with animals treated with vehicle. Intimal hyperplasia in INZ-701-treated *ttw/ttw* mice was greatly reduced and close to that observed in INZ-701-treated WT animals (Figure 7d). Carotid sections were stained for Ki-67, a marker of cell proliferation and for αSMA, to confirm that VSMC proliferation contributed to neointimal thickening after ligation. WT and *ttw/ttw* mice that received vehicle exhibited extensive Ki-67 and αSMA staining in the neointima (Figure 7e,f). Conversely, little Ki-67 staining was observed in WT and *ttw/ttw* mice treated with INZ-701 (Figure 7e).

**Figure 8:**
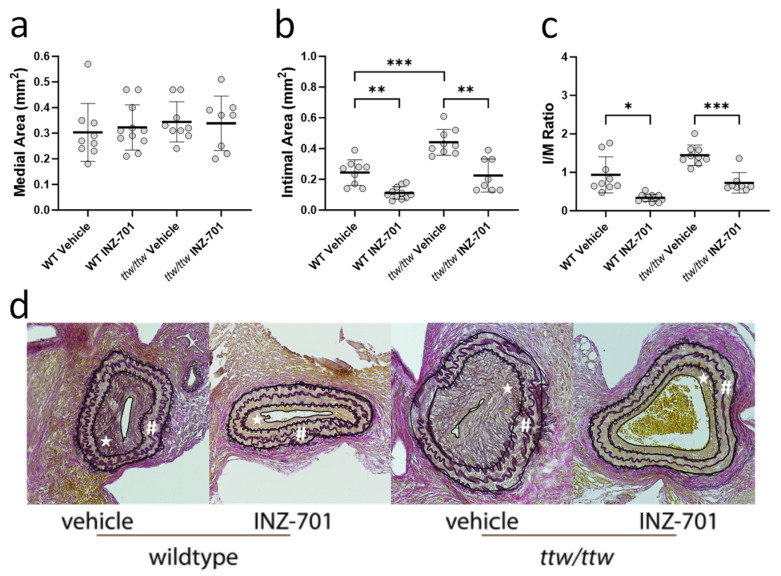
Effect of therapeutic treatment with INZ-701 on neointima formation in WT and *ttw/ttw* mice. Treatment of mice with either vehicle or INZ-701 (10 mg/kg) started on day 7 after ligation of left carotid artery. Carotid arteries were collected on day 14th after ligation and fixed in formalin. Morphometric analysis of elastin-stained sections was performed to determine medial (**a**) and intimal (**b**) areas, and the intima/media ratio (**c**). Elastin von Gieson’s staining of carotid arteries cross sections from vehicle- or INZ-701-treated WT and *ttw/ttw* mice (**d**). From outside to inside, external elastic lamina, internal elastic lamina, and luminal boarder are circled for better visualization in (**d**). The area between external elastic lamina and internal elastic lamina displays the medial area (#), the area between internal elastic lamina and luminal boarder displays the intimal area (star). Values are presented as the mean ± SD, *n* = 8–11, * *p* < 0.05, ** *p* < 0.01, *** *p* < 0.001 (one-way ANOVA).

To evaluate therapeutic effectiveness, INZ-701 treatment was initiated 7 days post ligation, when neointimal hyperplasia became evident (Supplemented Figure S3). The medial area, between the external and internal lamina, remained constant in all groups of mice (Figure 8a). Therapeutic treatment with INZ-701 led to a significant reduction in the neointimal area in WT and *ttw/ttw* mice when compared with vehicle-treated mice (Figure 8b,d). The I/M ratio of both INZ-701-treated WT and *ttw/ttw* mice was significantly decreased compared to the ratios observed in WT and *ttw/ttw* mice treated with vehicle (Figure 8c).

## 4. Discussion

VSMCs can exhibit remarkable phenotypic plasticity [24]. In a healthy vessel, VSMCs maintain a contractile phenotype, which is characterized by a low proliferation rate and diminished protein synthesis [23]. Under pathological conditions, VSMCs can de-differentiate and acquire different phenotypes including osteoblast-like, foam cell-like, and macrophage-like phenotypes [25]. These de-differentiated VSMCs contribute to the vascular wall inflammation, vascular calcification, and formation of the lipid-rich caps seen in atherosclerotic lesions [25]. In diseases associated with vessel wall thickening caused by neointima formation, VSMCs undergo a phenotypic switch to develop a synthetic phenotype [23], which is characterized by increased proliferation, migration, and deposition of the extracellular matrix [23]. Gene expression analysis indicated that expression of *Enpp1* and *CD73* ectonucleotidases was not affected by VSMC phenotype switching. However, the expression of *CD39* was significantly lower in synthetic compared to contractile VSMCs. In the cardiovascular system, CD39 is the main enzyme accountable for almost the entire ectonucleotidase activity at the inner vascular surface [26]. It is expressed on platelets [27], different subsets of white blood cells [27,28], endothelial cells [29], and smooth muscle cells. Due to its anti-thrombotic [30], anti-inflammatory [31,32,33], and anti-hypoxic activities [34], CD39 has been implicated in thromboregulation [26] and organ protection following ischemia reperfusion [35]. Different pathological conditions can dramatically modulate the expression of vascular CD39. Hypoxia causes the significant upregulation of CD39 in vessel walls [36], whereas endothelial CD39 expression and activity are markedly reduced in patients with idiopathic pulmonary hypertension [37]. In the latter condition, CD39 decrease was most pronounced in vessels with severe vascular remodeling [37]. Roy C. et al. studied the effect of blood pressure on the expression of arterial CD39 in spontaneously hypertensive rats and mice with angiotensin II-induced hypertension [38]. Compared to normotensive animals, hypertension resulted in the decrease in CD39 expression in conductance and resistance arteries with conductance arteries more affected [38]. In a mouse model of atherosclerosis, endothelial CD39 expression was lost in the vessel areas affected by plaques and CD39 deficiency led to accelerated atherosclerosis [39]. Interestingly, in this study, expression of CD39 was tightly regulated by the blood flow [39]. Partial cessation of flow by ligation of mouse carotid arteries acutely decreased expression of CD39 mRNA in the endothelium of ligated arteries but not in the control non-ligated arteries [39]. Similarly, CD39 expression in venous and arterial endothelial cells was significantly lower under static conditions than in conditions of laminar flow [39]. Hence, cessation of blood flow can dramatically downregulate CD39 expression and can thereby potentially affect the degradation of extracellular ATP and ADP.

Recently, it was reported that treatment of iPSC-derived smooth muscle cells with recombinant ENPP1 and ATP inhibited proliferation through the generation of AMP [18]. Herein, we extended these observations and demonstrated that extracellular AMP, produced from co-treatment with ENPP1 and ATP, can be further metabolized to adenosine by CD73 expressed on primary VSMCs of the synthetic phenotype. Our results indicate that CD73-dependent degradation of AMP to adenosine was required for the anti-proliferative effect and of the ENPP1 and ATP treatment. Moreover, adenosine by itself was capable of inhibiting cell proliferation. Adenosine generated by VSMCs activated Gs-coupled A_2a_ and A_2b_ adenosine receptors, which provoked an increase in intracellular cAMP and led to PKA activation. Previous in vitro and in vivo studies confirmed that activation of this signaling pathway using activators of adenylate cyclase [40], PDE inhibitors [41], or direct PKA activators [41,42,43,44] abrogated smooth muscle cell proliferation. Likewise, experiments have shown that activation of A_2a_ or A_2b_ receptors with selective adenosine receptor agonists, or in genetically modified mice [45], inhibited smooth muscle cell proliferation in vitro [46,47,48,49] and protected against neointimal hyperplasia in vivo [50,51,52].

The role of extracellular ectonucleotidases in vascular wall homeostasis has been studied in different models of vascular injury. Enhancement of ATP hydrolysis by administration of recombinant apyrase inhibits in-vein thrombosis and intimal growth in a mouse model of vein graft failure [53]. CD39 protects against pathological vascular remodeling and is central for normal vascular homeostasis [54]. In addition to ATP hydrolysis, CD39 has an ADP-hydrolyzing activity, which mediates its potent anti-platelet effect [30]. It was demonstrated that overexpression of CD39 decreases smooth muscle cell proliferation and neointima formation in rat iliac artery following balloon injury [55]. In a mouse model of wire-induced injury, treatment with soluble human CD39 reduced deposition of activated platelets and suppressed neointimal growth in injured femoral arteries [56].

CD73 hydrolyzes AMP, which is derived from ATP and ADP, to produce adenosine. Mice lacking CD73 demonstrated greatly exacerbated neointima formation in a wire injury model. Treatment of these mice with an A_2a_ receptor agonist restored adenosine-mediated signaling and prevented accelerated neointimal hyperplasia [50]. Unlike CD39, ENPP1 does not hydrolyze ADP and, therefore, lacks potent anti-platelet activity. Mice with monoallelic or biallelic ENPP1 deficiency developed neointimal thickening in response to carotid ligation [19]. The enhanced neointimal hyperplasia was associated with dysregulated smooth muscle cell function and increased expression of C/EBP homologous protein—a mediator of ER stress [19]. Recently, Y. Nitschke et al. examined the role that ENPP1 plays in arterial remodeling in *ttw/ttw* mice. These mice carry a biallelic nonsense mutation in *Enpp1* allele, which results in production of an enzymatically inactive truncated form of the protein [20]. Similar to CD73- and ENPP1-deficient mice, *ttw/ttw* mice developed thicker neointima in response to carotid ligation when compared to wild-type controls [18]. Both prophylactic and therapeutic treatments with recombinant human ENPP1-Fc significantly attenuated neointimal growth in *ttw/ttw* mice without affecting the tunica media [18]. INZ-701 is a recombinant human ENPP1-Fc fusion protein, which is currently being evaluated in clinical trials to treat patients with generalized arterial calcification of infancy (GACI) and autosomal recessive hypophosphatemic rickets type II (ARHR2), also known as ENPP1 deficiency [21]. Herein, we evaluated the effect of INZ-701 on neointimal formation in *ttw/ttw* and wild-type mice. Consistent with the data published by Nitschke et al. [18], neointima hyperplasia promoted by carotid artery ligation was more pronounced in *ttw/ttw* mice than in wild-type control animals. Prophylactic as well as therapeutic dosing with INZ-701 resulted in a significant reduction in intimal area in *ttw/ttw* mice. Interestingly, INZ-701 treatments reduced the formation of neointima in wild-type mice as well. Moreover, tunica media were also smaller in wild-type mice which received INZ-701 preventively. Staining for Ki-67, a biomarker of cell proliferation, was restricted to neointima only, with no Ki-67 observed in tunica media of wild-type or *ttw/ttw* mice. This result suggests that there is no proliferation of VSMC in this area. In addition to being a potent vasodilator, adenosine can have anti-hypertrophic activity. Thus, it was reported that adenosine inhibits protein synthesis and deposition of collagen in different cell types [57], including VSMCs [58]. It is therefore plausible that in wild-type mice, INZ-701 supplements the endogenous ENPP1 activity present in medial VSMCs. Of note, that unlike endogenous ENPP1 which is membrane bound, INZ-701 is a soluble form of the protein that distributes widely in tissues. This augmented local bioavailability of adenosine can promote vasodilation and reduce protein synthesis, which can potentially affect the tunica media morphology.

## 5. Conclusions

The work described herein demonstrated that CD73 expressed by VSMCs is critical for the production of adenosine from extracellular AMP and for the anti-proliferative effects of ENPP1 metabolism of ATP. The data indicate that adenosine generated by VSMCs engages Gs-coupled adenosine A_2a_ and A_2b_ receptors to activate the PKA signaling pathway (Supplementary Figure S5). While most of this data were generated using primary VSMCs, additional experiments in mice with genetically or pharmacologically inactivated CD73 and adenosine receptors are needed to further strengthen these initial findings. Finaly, in the carotid artery ligation model, treatment with recombinant ENPP1 attenuates neointimal thickening not only in Enpp1-deficient animals but also in wild-type mice. This enzyme enhancement therapy approach can suggest that a certain threshold of local adenosine is required to attenuate the neointimal proliferation. In addition to its known effect of generating the inhibitor of calcification, PP_i_, these findings provide insights into the mechanism of vaso-protection mediated by ENPP1, underline the role of ENPP1 as a mediator of purinergic signaling in the cardiovascular system (Supplementary Figure S5), and support further evaluation of ENPP1 in diseases with dysregulated smooth muscle cell function through the adenosine signaling pathway.

## 6. Patents

F.R. and Y.N. hold a patent on compositions and methods for treating allograft vasculopathy, Moyamoya disease, Moyamoya syndrome and intimal proliferation (WO/2021300261).

## Data Availability

Data are included in the article and Supplementary Materials.

## Supplementary Materials

The following supporting information can be downloaded at: https://www.mdpi.com/article/10.3390/cells13131128/s1.
