# Supplementary material for: Inhibition of Vascular Smooth Muscle Cell Proliferation by ENPP1: The Role of CD73 and the Adenosine Signaling Axis
Source: Cells. 2024 Jun 29;13(13):1128. doi: 10.3390/cells13131128 (PMC11240470; doi:10.3390/cells13131128)

Supplemental figures

Figure S1 (Supplemental). Characterization of contractile and synthetic VSMCs. VSMCs were differentiated towards synthetic or contractile phenotypes. Expression of the contractile biomarkers including smooth muscle myosin heavy chain (SM-MHC) and smooth muscle calponin (SM-Calponin) was analyzed by qPCR and Western Blot (**a**). For cell proliferation assays (**b**) synthetic and contractile VSMCs that had been starved for 24 hours in basal media were cultured in media containing FBS for 3 days. Cell proliferation was evaluated by BrdU incorporation. Values are presented as the mean ± SD, n=4-6, ***p<0.001, (t-test).

Figure S2 (Supplemental). Effect of AB680 on the Enpp1 enzymatic activity. IMA2a (1000 ng/ml) was incubated for 5 min with increasing concentrations of AB680 followed by determination of IMA2a enzymatic activity, n=1.

Figure S3 (Supplemental). Effect of Inosine on VSMCs proliferation. Synthetic VSMCs were starved for 24 hours in basal media. Cells were then cultured for 72 hours in basal media supplemented with 5% FBS in the presence or absence of inosine. Cell proliferation was evaluated by BrdU incorporation. Values are presented as the mean ± SD, n=8.

Figure S4 (Supplemental): Determination of the starting point for therapeutic treatment protocol. For determination of starting point, medial (**a**) and intimal (**b**) area and I/M ratio (**c**) of *ttw/ttw* mice ligated for 7, 10 and 14 days were evaluated. Formation of neointima in ttw/ttw mice was confirmed on day 7 post ligation. Values are presented as the mean ± SD, n=3-11, *p<0.05, ***p<0.001 (one-way ANOVA).

Figure S5 (Supplemental): Proposed mechanism of anti-proliferative effect of ENPP1. The transmembrane ecto enzyme ENPP1 converts extracellular ATP to AMP and PP_i. AMP is further hydrolyzed by CD73 (ecto-5-exonucleotidase) to adenosine and P_i. CD73 expressed by VSMCs is critical for production of adenosine from extracellular AMP. Adenosine generated by VSMCs activated Gs-coupled adenosine receptors A_{2A} and A_{2B}, which leads to an activation of adenylate cyclase, followed by an increase in intracellular second messenger cAMP and PKA activation. Activation of cAMP-PKA signaling pathway inhibits VSMC proliferation. (Created with BioRender.com)

Figure S1 (Supplemental)

a

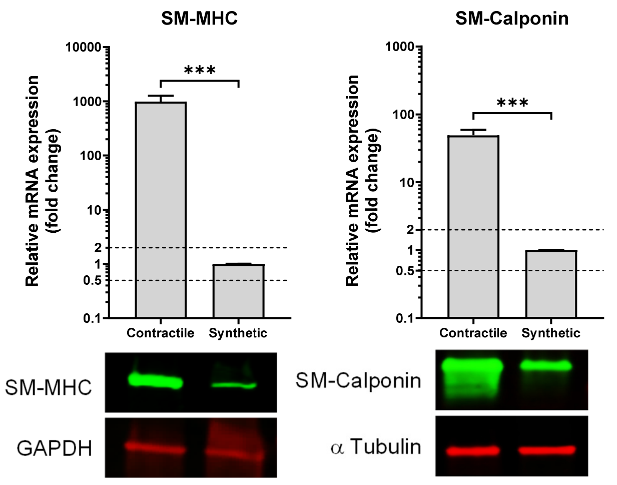

b

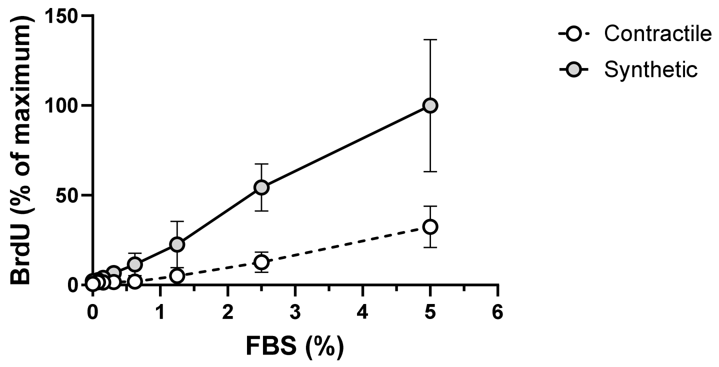

Figure S2 (Supplemental)

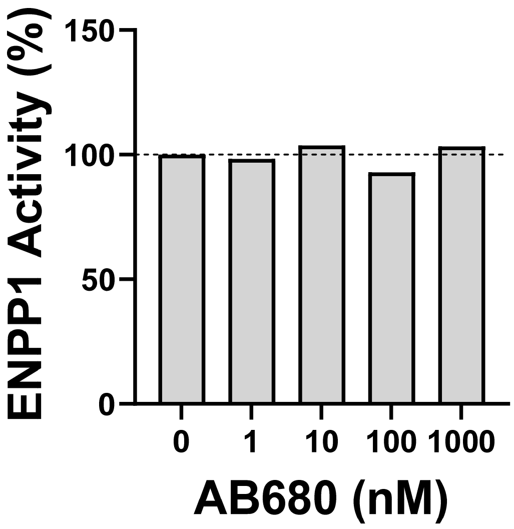

Figure S3 (Supplemental)

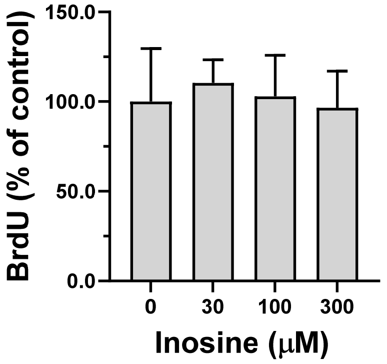

Figure S4 (Supplemental)

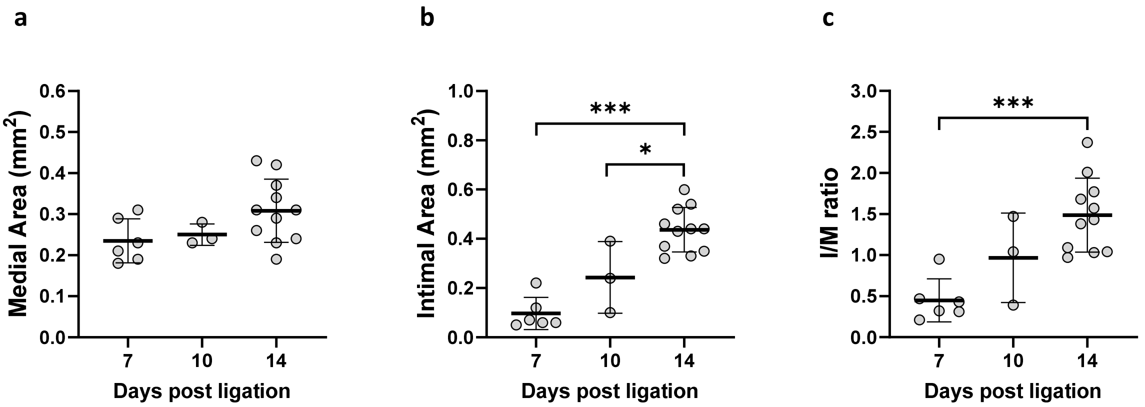

Figure S5 (Supplemental)

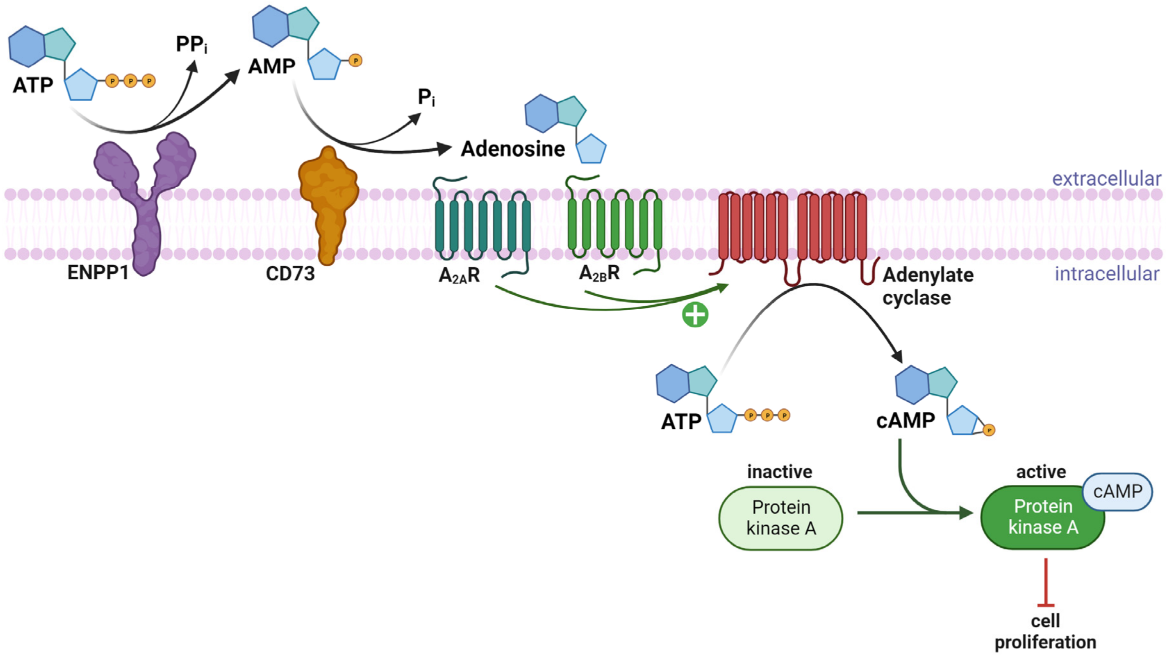

Supplement: Supplementary file 1 [file cells-13-01128-s001.zip › cells-3004630-supplementary.pdf]
